# Fairness Correction in COVID-19 Predictive Models Using Demographic Optimization: Algorithm Development and Validation Study

**DOI:** 10.2196/78235

**Published:** 2026-02-03

**Authors:** Naman Awasthi, Saad Abrar, Daniel Smolyak, Vanessa Frias-Martinez

**Affiliations:** 1 Department of Computer Science, University of Maryland, 8125 Paint Branch Ave, College Park, MD, 20742, United States, 1 2402806921; 2 College of Information Studies, University of Maryland, College Park, MD, United States

**Keywords:** COVID-19 forecasting, deep learning model, fairness, time series model, regression

## Abstract

**Background:**

COVID-19 forecasting models have been used to inform decision-making around resource allocation and intervention decisions, such as hospital beds or stay-at-home orders. State-of-the-art forecasting models often use multimodal data, including mobility or sociodemographic data, to enhance COVID-19 case prediction models. Nevertheless, related work has revealed under-reporting bias in COVID-19 cases as well as sampling bias in mobility data for certain minority racial and ethnic groups, which affects the fairness of COVID-19 predictions across racial and ethnic groups.

**Objective:**

This study aims to introduce a fairness correction method that works for forecasting COVID-19 cases at an aggregate geographic level.

**Methods:**

We use hard and soft error parity analyses on existing fairness frameworks and demonstrate that our proposed method, Demographic Optimization (DemOpts), performs better in both scenarios.

**Results:**

We first demonstrate that state-of-the-art COVID-19 deep learning models produce mean prediction errors that are significantly different across racial and ethnic groups at larger geographic scales. We then propose a novel debiasing method, DemOpts, to increase the fairness of deep learning–based forecasting models trained on potentially biased datasets. Our results show that DemOpts can achieve better error parity than other state-of-the-art debiasing approaches, thus effectively reducing the differences in the mean error distributions across racial and ethnic groups.

**Conclusions:**

We introduce DemOpts, which reduces error parity differences compared with other approaches and generates fairer forecasting models compared with other approaches in the literature.

## Introduction

### Background

Forecasting the number of COVID-19 cases, hospitalizations, or deaths is crucial to inform decision-making. For example, COVID-19 forecasts can be used by hospitals to evaluate medical needs and required resources, such as supplies or beds, or by public health officials to inform closure policies at various geographical scales. In the United States, COVID-19 forecasts have been used at the state and county levels to inform social distancing or masking, such as the publicly available forecasts on the COVID-19 Forecast Hub that the Centers for Disease Control and Prevention (CDC) has routinely used in their communications [1,2].

Related work over the past 4 years has shown a diverse variety of COVID-19 forecasting approaches [3-10, 11] using datasets such as the New York Times (NYT), Johns Hopkins University, COVID-19 Community Vulnerability Index, Google, and Apple [12-16], among others. Most publications focused on COVID-19 case prediction have reported results around the accuracy of the models, that is, minimizing the difference between the predicted cases and the actual number of cases reported. Nevertheless, previous work has shown that the accuracy of COVID-19 predictions can depend on various social determinants, including race or ethnicity [17], income, or age [18], revealing worse performance for protected attributes and pointing to a lack of COVID-19 predictive fairness that can affect resource allocation and decision-making. This lack of predictive fairness might be related to bias in the datasets used to train the model, that is, bias in COVID-19 case reporting [19] or bias in mobility data [20].

Given the presence of bias in the training datasets frequently used by COVID-19 forecast models, and previous work demonstrating that COVID-19 prediction accuracy can vary across social determinants, it becomes critical to devise methods to prevent data biases from percolating into the COVID-19 forecasts to guarantee fair decision-making based on case predictions. In this paper, we focus on in-processing bias mitigation approaches given their scarcity in the COVID-19 literature and propose Demographic Optimization (DemOpts), a debiasing method designed to achieve COVID-19 case prediction error parity across racial and ethnic groups in the context of deep learning models, that is, guarantee that county prediction errors are not significantly different across racial and ethnic groups. Although there exists a diverse set of COVID-19 predictive approaches, we focus on deep learning models because these are the most frequently used models in the machine learning community [21], and narrow down our choice to transformer-based architectures because they are state-of-the-art in time series predictions [22].

The main objective of DemOpts is to improve the fairness of the COVID-19 case predictions at the county level by achieving error parity in a regression setting [17]. DemOpts proposes a novel debiasing approach that leverages county racial and ethnic data during training to modify conventional deep learning loss functions to penalize the model for statistically significant associations between the predictive error and the race or ethnicity distribution of a county. Our main contributions are:

- We present DemOpts, a novel debiasing method for deep learning architectures that attempts to increase the fairness of the COVID-19 county case predictions by achieving error parity, that is, guaranteeing that prediction errors are similar across racial and ethnic groups. The DemOpts architecture is designed to optimize error parity across race and ethnicity using a novel multilabel approach that allows each county to be characterized by its own racial and ethnic group distribution during the debiasing process, instead of by a unique label.
- We propose a novel evaluation protocol for the COVID-19 context, and we show that (1) state-of-the-art COVID-19 county case prediction models based on transformer architectures with no debiasing approach lack error parity, that is, prediction errors are statistically significantly different across racial and ethnic groups, (2) DemOpts applied to transformer-based architectures improves the error parity of the prediction models, increasing the similarity between mean prediction errors across racial and ethnic groups, and (3) the DemOpts debiasing approach performs better than state-of-the-art debiasing methods for regression settings.

While COVID-19 research was particularly prominent from 2020 to early 2024, challenges related to data biases and sampling issues in predictive modeling remain highly relevant. Our approach, leveraging the regression fairness model DemOpts, provides a robust framework to address these challenges. As future pandemics and public health crises arise, similar issues will persist, making our contribution valuable for ensuring fairness and reliability in predictive models.

### Literature Review

#### Deep LearningBased Forecasting Models

Deep learning models have started to become popular in time series prediction tasks. The available methods include (1) autoregressive models, such as Long Short-Term Memory or Gated Recurrent Network [23]; (2) graph-based neural networks, such as graph attention networks [24], Spatio-temporal Graph Convolutional Network [25], neighbor convolution model [26], or graph convolutional network; and (3) transformers, including Logarithmic Sparse Transformer [27], Informer [28], Autoformer [29], Frequency Enhanced Decomposed Transformer [30], Pyramidal Attention–based Transformer [31], and Patch Time Series Transformer [32]. In this paper, we specifically focus on the temporal fusion transformer (TFT) architecture [22], since it allows us to easily incorporate exogenous variables (eg, mobility data) as well as static variables (eg, demographic data) on top of the COVID-19 time series.

#### Bias in Mobility and COVID-19 Data

The COVID-19 epidemic was closely monitored and had extensive data available about the counts of cases, hospitalizations, and deaths, as well as fine-grained information about mobility of people, policy implementations, vaccinations, and so on. Reducing the impact of mobility data or COVID-19 case bias in COVID-19 case predictions, as we do in this paper, is of critical importance to support decision-making processes focused on resource allocation during pandemics, to reduce harm and guarantee that decisions are fair and just across racial and ethnic groups. Human mobility data has been used to characterize human behaviors in the built environment [33-37], for public safety [38,39], during epidemics and disasters [40-45], as well as to support decision-making for socioeconomic development [46-53]. During the COVID-19 pandemic, human mobility has played a central role in driving decision-making, acknowledging the impact of human movement on virus propagation [7,9,10,18,54]. Previous work has revealed sampling bias in mobility data collected via mobile apps, with Black and older individuals being underrepresented in the datasets [20], and has exposed biases in COVID-19 forecasting models [55,56]. COVID-19 underreporting bias has been discussed in the literature [57-59] and points to multiple causes, including inadequate testing across certain minority groups or a lack of consistency in reporting race and ethnicity for COVID-19 cases [19].

#### Fairness Metrics and Fairness Corrections

Transformer-based COVID-19 case forecast models require the use of fairness metrics for regression settings, given that the loss optimization process in gradient-based deep learning architectures uses real-number predictions instead of classes.

Agarwal et al [60], Fitzsimons et al [61], and Gursoy and Kakadiaris [17] outline the different aspects of fairness in regression settings and propose a set of fairness metrics for regression-type models. For this paper, we use the error parity metric proposed in [17]. Error parity requires error distributions to be statistically independent of racial and ethnic groups. We expand this definition and relax the statistical significance requirement to be able to also evaluate whether the proposed DemOpts method can at least reduce the differences in error distributions across racial and ethnic groups, even when they are still statistically significantly different. To correct for bias and unfair performance in deep learning models, researchers have used preprocessing [62,63] and in-processing correction approaches [64-67]. Preprocessing approaches focus on creating a better input for learning deep neural network models by removing bias from the datasets [62,63], and there have been successful efforts focused on debiasing underreporting COVID-19 datasets to estimate actual cases or deaths before they are fed into predictive models [68,69]. On the other hand, in-processing approaches to improve the fairness of deep learning models, like the one we use in this paper, focus on the model and its regularization, usually adding a bias correction term in the loss function [65,67]. In this paper, we will compare our proposed debiasing approach against 3 state-of-the-art methods for debiasing in regression settings, which are individual fairness correction [70], group fairness correction [70] (both Lagrangian-based), and sufficiency [71]. Individual and group fairness calculate penalties by determining overestimations across different groups and weighting the loss by a factor proportional to the overestimations, while sufficiency-based regularizers propose to make the loss independent of sensitive data attributes by simultaneously training a joint model and subgroup-specific networks to achieve fair predictions [71].

## Methods

### Proposed DemOpts

Our modeling focus is on deep learning models, which are the most frequently used approach for COVID-19 county case forecasts in the machine learning community [21]. We specifically focus on the TFT model introduced in [22] for several reasons. First, this model is state-of-the-art in interpretable time series prediction [22]. Second, this model allows for the use of static reals as input to the model (ie, attributes that do not change over the duration of the training process, such as demographic percentages or population statistics). Third, the model works well with time-dependent features, including COVID-19 cases or mobility data, whereby past data influences future statistics.

DemOpts is an in-processing algorithm that modifies the standard training procedure for deep learning models at the loss computation stage. The algorithm modifies conventional loss functions to penalize the model for any statistically significant association (*P*<.005) between the county prediction loss (error) and the county’s racial and ethnic groups. In other words, DemOpts performs a race-based correction on the error to account for county demographic, racial, and ethnic distributions.

**Figure 1.**
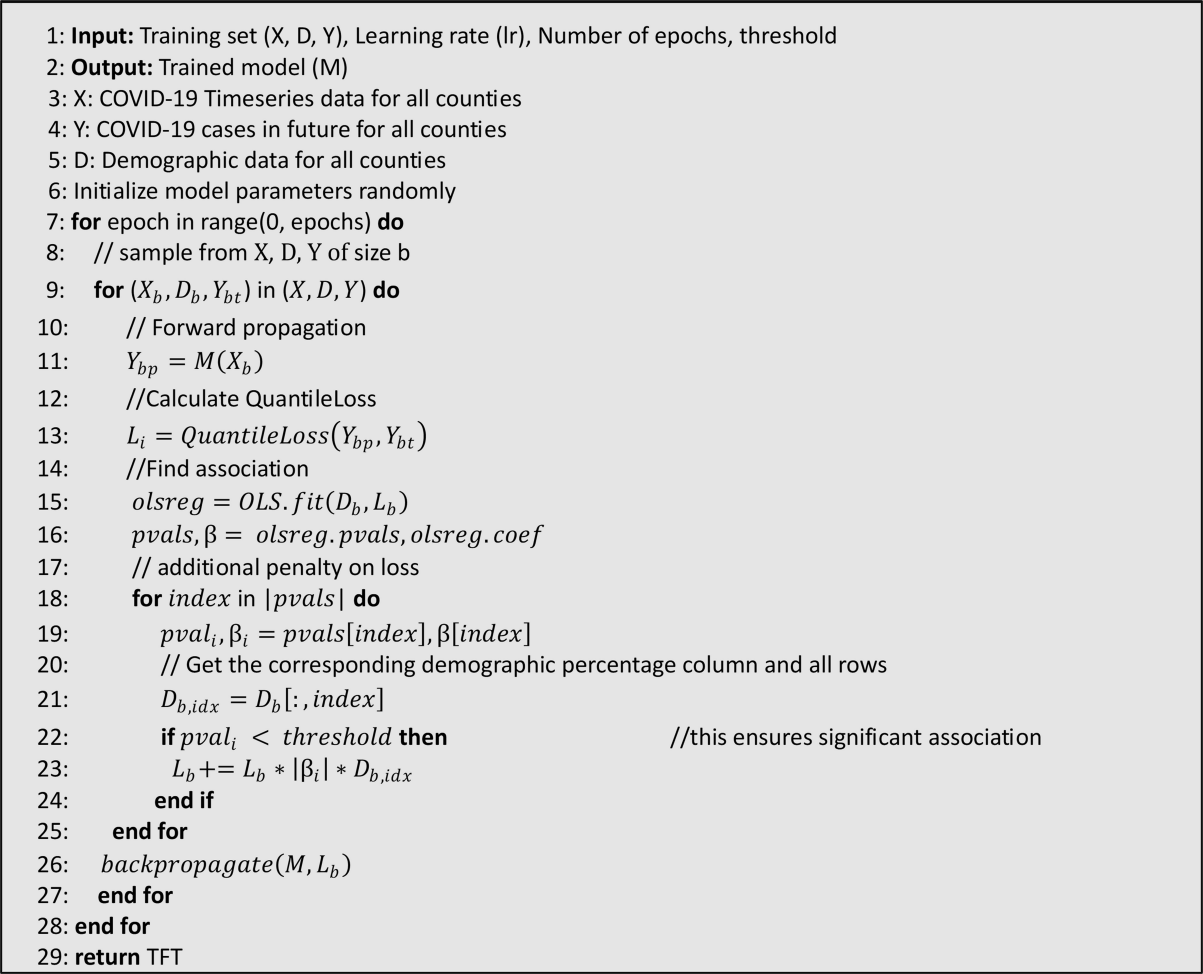
Algorithm: Demographic Optimization (DemOpts). TFT: Temporal Fusion Transformer.

**Figure 2.**
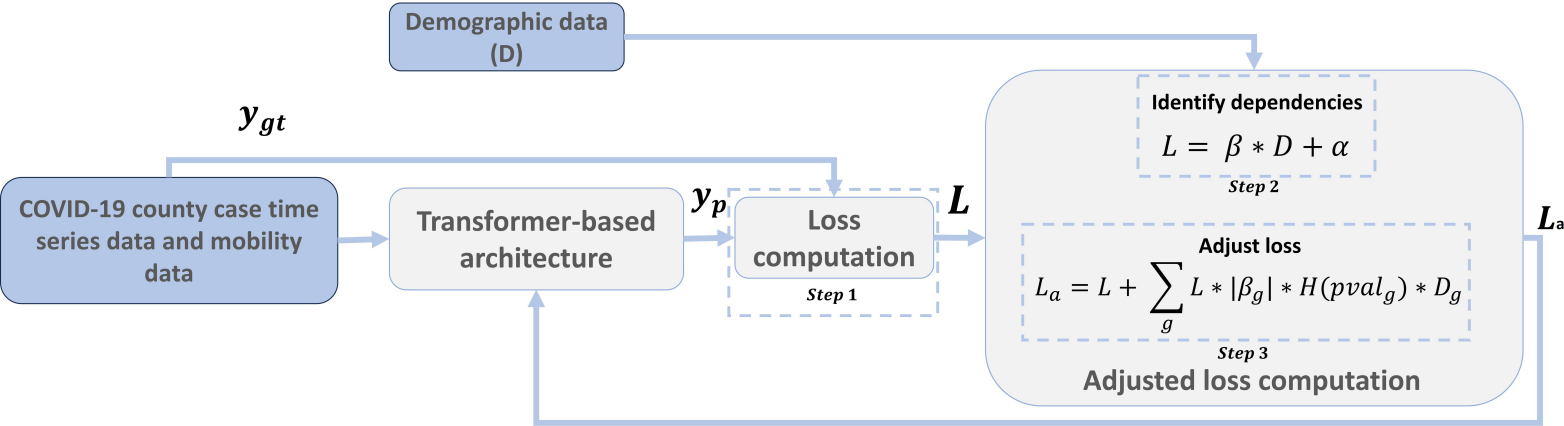
Flow diagram for the Demographic Optimization (DemOpts) method.

The algorithm can be divided into 3 steps (refer to Figure 1, Figure 2, and “S.1 DemOpts Method” in Multimedia Appendix 1 for mathematical details).

#### Step 1: Calculate Loss

We use quantile predictions, as standard in COVID-19 forecasting literature [2,72], instead of point-value predictions. Quantile predictions are measured for 7 quantiles (0.02, 0.1, 0.25, 0.5, 0.75, 0.9, and 0.98) to gain insights into the uncertainty ranges and CIs of the COVID-19 county case predictive models. When using quantile predictions, the error is computed using quantile loss, also known as pinball loss (PBL), and defined as follows:

$PBL_{q}\left(y_{ip},y_{i}\right)=\left\{ \begin{array}{l} q*\left(y_{i}-y_{ip}\right)\;\;\;\;\;\;\;\;\;\;\;\;\;\;\;\;\;\;\;\;\;\;\;\;\;\;\;\;\;\;\;\;\;\;\;\;\;\;\;\;\;\;\;\;\;\;\;\;\;\;\;\;if\;\;\;y_{i}\geq y_{ip}\\ \left(q-1\right)*\left(y_{i}-y_{ip}\right)\;\;\;\;\;\;\;\;\;\;\;\;\;\;\;\;\;\;\;\;\;\;\;\;\;\;\;\;\;\;\;\;\;\;\;\;\;\;\;\;\;\;if\;\;\;y_{i}<y_{ip} \end{array}\right.$

For quantile *q*, the PBL for the prediction of a given input *X_i_* is *PBL_q_(y_ip_, y_i_*), where *y_i_* is the ground truth, and *y_ip_* is the predicted value. The average over all quantiles can be represented as $PBL\left(y_{ip},\;y_{i}\right)\;=\;\frac{1}{\left|q\right|}\sum_{q}PBL_{q}\left(y_{ip},\;y_{i}\right)$.

#### Step 2: Identify Dependencies Between Prediction Errors, Race, and Ethnicity

To achieve error parity, that is, mean errors being independent of racial and ethnic population distributions, we determine the relationship between errors and race and ethnic distributions. For that purpose, DemOpts fits a regression model between the prediction losses PBL (y_ip_,y_i_) across data points *i* and their corresponding county race and ethnicity distribution for each race D_i_:


$$PBL\left(y_{ip},y_{i}\right)=\;\beta *D_{i}\;+\;\alpha \;\;\;\;\;\;\;\;with\;\;\;\;\;\;\;D_{i}\;=\;\left[d_{1},d_{2},d_{3},d_{4},\;lookahead\right]$$

where d_i_ are the corresponding county demographic features extracted from the US census data (represented as the percentage of each racial and ethnic group of the county for datapoint i), and lookahead refers to the number of days into the future the COVID-19 case prediction was generated for. In matrix representation:


$$PBL\left(Y_{ip},Y_{i}\right)=\beta *D+\alpha$$

Once the regression model is fit, both regression coefficients (β) and their statistical significance (*P* value) are passed on to Step 3 to modify the adjusted loss and attempt to decouple race from the errors (loss).

#### Step 3: Adjust the Loss

DemOpts modifies the conventional loss of deep learning models by adjusting for racial or ethnic bias in the error, that is, the loss is increased whenever a statistically significant regression coefficient for a race or ethnicity is found in Step 2 (with *P* value threshold=.005). By increasing the loss, DemOpts attempts to reduce the association between errors and race. Specifically, the loss is adjusted by the product of the original loss PBL (y_ip_,y_i_), the percentage race or ethnicity D_j_ that holds a significant relationship with the error, and its coefficient β_j_ in absolute value:


$$L_{adj}=PBL\left(y_{ip},y_{i}\right)+\sum_{j}H\left(pval_{j}\right)\left(\left|\beta_{j}\right|*D_{j}*L\right)\;\;where$$

$$H\left(x\right)=\left\{ \begin{array}{l} 1\;\;\;\;\;\;\;\;\;\;\;\;\;\;\;\;\;\;\;\;\;\;\;\;\;\;\;\;\;\;\;\;\;\;\;\;\;\;\;if\;x\;<\;0.005\\ 0\;\;\;\;\;\;\;\;\;\;\;\;\;\;\;\;\;\;\;\;\;\;\;\;\;\;\;\;\;\;\;\;\;\;\;\;\;\;\;if\;x\;\geq \;0.005 \end{array}\right.$$

### Evaluation Protocol

In this section, we present a novel evaluation protocol to assess changes in fairness for TFT forecasting models when debiasing approaches, including DemOpts, are applied. We first describe the TFT COVID-19 county case prediction model we use, and the different debiasing approaches we evaluate on that prediction model. Next, we describe the error parity metrics we use to evaluate the fairness of each prediction model, and finally, we present the approach to analyze whether DemOpts improves the error parity metrics when compared to other state-of-the-art debiasing approaches for regression settings.

#### Predictive Model and Debiasing Approaches

We use the TFT with the conventional PBL function (PBL is the standard metric for reporting model performance in CDC Forecast Hub [2]) as our baseline model (TFT_Baseline_) to predict the number of COVID-19 county cases for a given day.

Input data to the TFT model includes past COVID-19 cases per county, mobility data from SafeGraph, and race and ethnicity data for the county. We also train and test another TFT enhanced with the DemOpts debiasing method, TFT_DemOpts_, that adjusts the loss computation to attempt to eliminate or reduce the dependencies between error and race to achieve error parity. In addition, we train and test 3 more TFTs enhanced with state-of-the-art debiasing methods for regression settings, namely, individual fairness TFT_Individual_ [70], group fairness TFT_Group_ [70], and the sufficiency-based regularizer TFT_Sufficiency_ [71]. Individual and group fairness methods calculate penalties by determining overestimations across different groups and weighting the loss by a factor proportional to the overestimations, while the sufficiency-based regularizer trains a joint model and group-specific networks to achieve fair predictions. We replicate their methodology and adapt it to the forecasting setting by keeping TFT as the common network.

#### Measuring Model Fairness

We choose error parity as our fairness metric [17], with a focus on evaluating whether the distribution of predictive errors at the county level is independent of county majority race and ethnicity, that is, prediction errors are not statistically significantly different across racial and ethnic groups. To measure the fairness of each of the models TFT_Baseline_, TFT_DemOpts_, TFT_Individual_, TFT_Group_and TFT_Sufficiency_, we propose a 2-step process.

##### Step 1: Associate Errors With County Race or Ethnicity

To carry out the fairness analysis, we need to associate the PBL error of each county with race and ethnicity labels. However, that would require access to race-stratified COVID-19 case data at the county level, which is unfortunately not available due to systemic data collection failures during the pandemic [73]. Hence, we propose to associate each county and its error with the majority race, that is, we label each county with the race or ethnicity that has the highest population percentage in that county. During the fairness analysis, we refer to majority White counties as the unprotected group and majority minority counties, such as Black or Hispanic, as the protected groups (details about the racial and ethnic groups considered in the evaluation are provided in the “Datasets” section).

In addition, we normalize each county’s PBL error by county population size. The normalization by county population allows us to scale the errors appropriately, since higher-population counties will have higher case counts and thus, higher-magnitude errors. Normalizing by population fairly compares the error per unit population of one county with another:


$$NormPBL\left(y_{pi},y_{ti}\right)=\frac{1000\cdot PBL\left(y_{p,i},y_{t,i}\right)}{pop_{i}}$$

where y_ti_ is the ground truth, y_pi_ is the predicted value, and pop_i_ is the county population.

We then calculate the average normalized PBL for each racial or ethnic group:


$$AvgNormPBL\left(y_{p},y_{t},g\right)=\frac{\sum_{i\in c_{g}}NormPBL\left(y_{pi},y_{ti}\right)}{\left|c_{g}\right|}$$

where g represents the racial or ethnic group and c_g_ is the set of all counties with as the majority group. This gives us the average normalized PBL for each demographic group.

##### Step 2: Compute Fairness Metric

Once PBLs have been calculated for each racial and ethnic group in the United States, we can compute the error parity, that is, the fairness metric focused on evaluating whether the prediction errors are different across race and ethnicity. We propose 2 metrics to measure the error parity of COVID-19 county case predictions: hard error parity and soft error parity.

### Hard Error Parity Metric

Model predictions exhibit hard error parity when no statistically significant differences exist between normalized mean case prediction errors (AvgNormPBL) across racial or ethnic groups. In other words, normalized mean PBL errors across counties of different racial and ethnic groups are similar and hence, not biased by race or ethnicity. To test for the hard error parity of a prediction model, we propose to run one-way ANOVA followed by post hoc Tukey honestly significant difference (HSD) tests between the normalized mean error distributions of all racial and ethnic groups. ANOVA tests are an adequate choice even in violation of normality for large sample sizes, and in the presence of unequal sample sizes with homogeneous variance; thus, we choose this parametric test due to its superior strength [74,75].

Rejecting the null hypothesis for ANOVA would point to significantly different mean error values across some racial or ethnic groups and to a lack of perfect hard error parity. The subsequent analysis of the post hoc Tukey HSD test would reveal the pairs of racial and ethnic groups whose mean error values are significantly different and the numerical difference. The Tukey test also highlights the pairs of racial and ethnic groups for which the mean error is not statistically significantly different, pointing to instances where hard error parity exists for that model.

### Soft Error Parity Metric

Instead of measuring the statistical significance of the relationship between county race labels and county errors, we propose to use the Accuracy Equity Ratio (AER) metric [76]. AER computes the ratio between the errors of the protected and unprotected groups as follows:


$$AER_{pg}=\frac{AvgNormPBL\left(y_{p},y_{t},pg\right)}{AvgNormPBL\left(y_{p},y_{t},unpg\right)}$$

where subscript pg indicates counties labeled as the protected group (majority minority counties). unpg indicates counties labeled as the unprotected group (White), and AvgNormPBL is the average of the normalized PBL across counties for a given racial group g (pg or unpg).

As defined, the AER metric goes from 0 to ∞. AER values in the range [0, 1] indicate comparatively lower normalized PBL for protected groups, which means the model predictions could be biased—have higher errors—for White majority counties; while AER values larger than one indicate that the model could be biased against the protected group, that is, the prediction errors are larger for counties with majority-minority groups. Values close to 1 indicate parity in error distribution between the protected group counties and the majority White counties. We claim that a predictive model achieves soft error parity for a given protected group when the AER value is close to 1, that is, the mean predictive error between that protected group and the White race is similar.

An alternative approach to assigning majority race or ethnicity would be to explore the associations between PBL errors and the distribution of racial and ethnic groups in a county (independent of COVID-19 cases, since that data are not available). Using a quantile regression, we can explore whether DemOpts eliminates significant associations between racial or ethnic percentages and the PBL errors, or at least reduces their magnitude. This approach removes the majority race requirement, but does not allow us to perform analyses with well-established fairness metrics in the literature, such as AER. Results are provided in the Multimedia Appendix 1.

### DemOpts Over State-of-the-Art

To assess whether DemOpts is a better debiasing approach than state-of-the-art methods, we need to compare the error parity metrics of the COVID-19 county case prediction model enhanced with the DemOpts method, TFT_DemOpts,_ against the error parity metrics of the same prediction model enhanced with the other debiasing approaches (individual TFT_Individual_, group TFT_Group_, or sufficiency TFT_Isufficiency_), as well as with the baseline COVID-19 county case prediction model without any debiasing approach, TFT_Baseline_. Next, we describe how we carry out this analysis for the hard and soft error parity metrics.

### Hard Error Parity

We computed the hard error parity metric for each of the COVID-19 county case prediction models, using one-way ANOVA and the post hoc Tukey HSD test. An exploration of the statistical significance of the mean error difference for each pair of racial and ethnic groups will reveal whether applying DemOpts to the COVID-19 case prediction model produces fewer instances of significant mean error differences than any of the other debiasing methods or the baseline. In other words, a decrease in the number of significantly different mean PBL errors between races would point to an achievement of hard error parity for more racial and ethnic groups than other state-of-the-art debiasing approaches or the baseline.

### Soft Error Parity

To assess whether DemOpts applied to a COVID-19 case prediction model has higher soft error parity than any of the other state-of-the-art debiasing approaches, we propose to compare the AER values for each protected race and ethnic group across the 5 models: TFT_DemOpts_, TFT_Individual_, TFT_Group_, TFT_Sufficiency_, and TFT_Baseline_. Since AER values represent the quotient between the normalized mean prediction errors of a protected race or ethnicity vs White counties, the model with AER values closer to 1 will be the approach with the highest soft error parity. To measure AER’s distance to 1, we compute the distance=|1-AER_race_| for each race and ethnic group, which represents the distance to a perfect soft parity error of 1. Distances closer to zero reveal better soft error parities.

### Datasets

In this section, we discuss the datasets we use in the DemOpts evaluation in the “Results” section. We train COVID-19 county case prediction models for the United States using COVID-19 case data, as well as mobility and demographic data. Mobility data has been used by previous work to inform case predictions via human mobility behaviors, under the assumption that the way people move might have an impact on the spreading of the epidemic. On the other hand, demographic data, either raw from the census or combined in different types of vulnerability indices, has also been shown to help predict COVID-19 prevalence, given the fact that COVID-19 has heavily affected vulnerable populations [59].

#### COVID-19 Case Data

**Figure 3.**
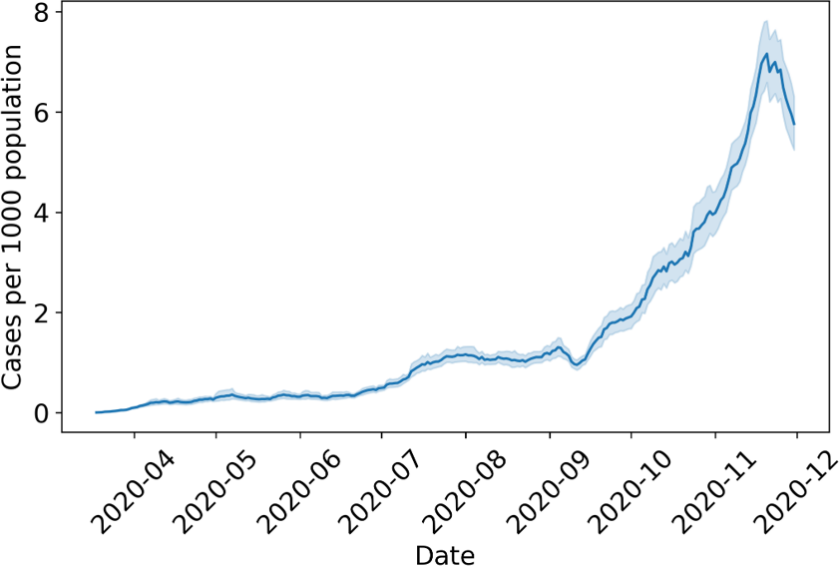
COVID-19 reported case counts per 1000 population across the United States.

We use the COVID-19 case data compiled by the NYT at the county level [12]. We account for delayed reporting by using the 7-day daily rolling average of COVID-19 cases (computed as the average of its current value and 6 previous days) instead of raw counts. Figure 3 charts the daily COVID-19 reported cases throughout the data collection period.

#### Mobility Data

SafeGraph open-sourced the mobility patterns of smartphone app users at the onset of the pandemic. These data points are curated by tracking the movements of millions of pseudonymized users via mobile app Software Development Kits (SafeGraph). Based on the data available, we use the daily origin-destination (OD) county-to-county flows [77]. OD flows represent the volume of trips between pairs of counties across the United States for each day. For OD flows, we only use SafeGraph inflow (ie, mobility into the county). The inflow mobility is measured as changes in volumes of flows with respect to a baseline of normal behavior computed by SafeGraph using mobility data from February 17, 2020, to March 7, 2020.

**Figure 4.**
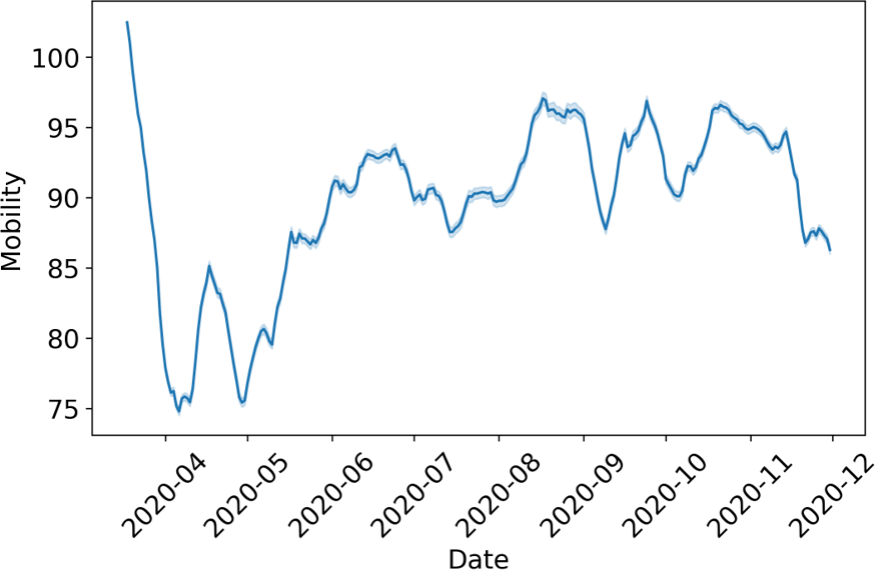
Mobility for all ethnic and racial groups.

Previous work has shown sampling bias in mobility datasets, revealing that not all races and ethnicities are equally represented due to variations in smartphone penetration rates [20,78]. It has also been shown that sampling bias in mobility data can negatively impact downstream tasks such as COVID-19 forecasting [56]. While the addition of mobility data could potentially help improve prediction accuracy and support better decision-making, it also introduces bias. Our empirical analysis of DemOpts aims to understand whether the debiasing method proposed in this paper can improve the fairness of COVID-19 county case predictive models when mobility data is used as input to the predictive model. Figure 4 shows the aggregate mobility data across the country. We see an initial drop in mobility in April (2020‐04), which corresponds to the first lockdown period. We then observed an increase in mobility a month later, which partially stabilizes after April.

#### Race and Ethnicity Data

**Figure 5.**
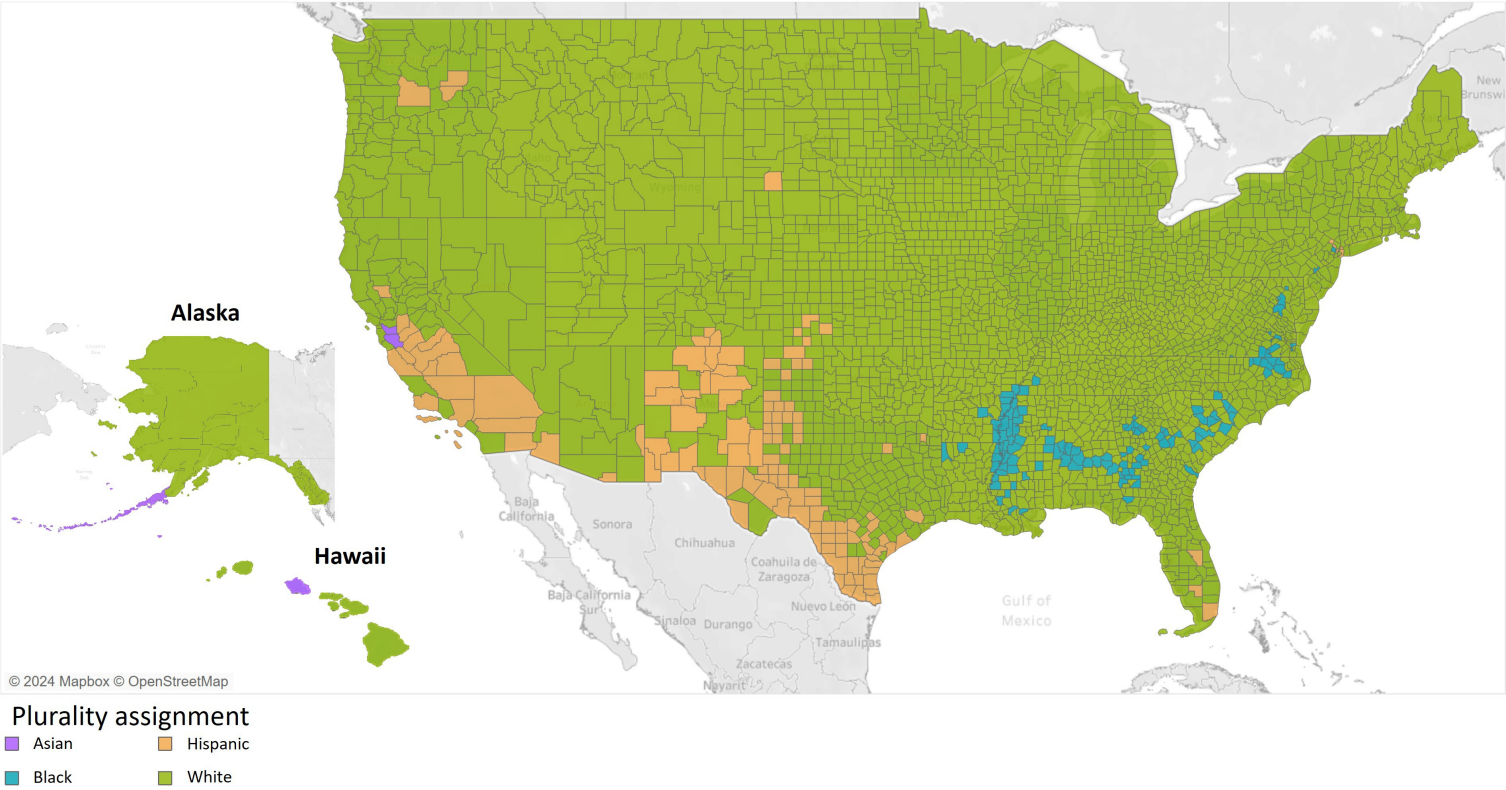
Counties and majority-based label. (Mapbox and OpenStreetMap were used to create this map [79,80,81].)

We retrieve the race and ethnicity data from each county in the United States from the 2019 5-year American Community Survey. This survey collects data annually from all 50 states, Puerto Rico, and Washington, DC. As described in Step 1 of the evaluation protocol, we associate each county and its errors with the majority race (ie, we label each county with the race or ethnicity that has the highest population percentage in that county). Following this procedure identifies 4 racial and ethnic groups for the majority of counties: Asian, Black, Hispanic, and White. Table 1 shows the distribution of US counties into these 4 racial and ethnic groups, and Figure 5 show color-coded maps with the majority racial or ethnic group for each county.

**Table 1.** Majority label counts.

Majority label	Count, n (%)
Asian	6 (0.194)
Black	127 (4.118)
Hispanic	126 (4.085)
White	2825 (91.601)

### Model Training

For evaluation purposes, we use COVID-19 case and SafeGraph mobility data from March 18, 2020, to November 30, 2020, for the training (207 days) and testing (49 days) of the TFT COVID-19 county case prediction models. The forecast task is the prediction of the number of COVID-19 cases for a given county from day X+1 to X+50, that is, the following 2 months (long-term forecasting with lookahead values from 1 to 50). Specifically, we train and test (1) the TFT_Baseline_, a TFT prediction model without a debiasing method; (2) the TFT_Individual_, TFT_Group_, and TFT_Sufficiency_, TFT prediction models with state-of-the-art debiasing methods; and (3) TFT_DemOpts_, a TFT prediction model enhanced with our proposed debiasing method. All 5 models are trained and tested for the same temporal range, and all are implemented using the PyTorch Forecasting library [82]. We limit the period of analysis to a time before COVID-19 vaccines were available, given that after that event, research has revealed a less clear relationship between mobility data and postvaccines COVID-19 case volumes [83]. We use the prediction errors (PBL) per racial and ethnic group to analyze and compare the hard and soft error parity of all trained models.

### Ethical Considerations

We used openly available datasets for mobility data (SafeGraph), COVID-19 case count (NYT), and demographic data (American Communities Survey). There was no human participant recruitment in this study, and thus we did not require institutional review board approval. All the datasets were aggregated at the county level and do not pose the risk of deanonymization.

## Results

### Hard Error Parity Results

ANOVA tests of the normalized mean PBL error distributions across racial and ethnic groups for each debiasing approach were all significant, pointing to a dependency between race and the normalized prediction errors.

Table 2 shows the *F* statistic and test significance for each of the prediction models with and without debiasing approaches. The significant ANOVA tests reveal that perfect hard error parity is not achieved by any of the debiasing methods. In other words, for some racial and ethnic groups, there exist statistically significant differences between their mean PBL prediction errors of different racial and ethnic groups; this effect occurs for the TFT_Baseline_ model as well as across all the other predictive models enhanced with a debiasing approach.

Nevertheless, post hoc Tukey HSD tests revealed interesting, nuanced results, showing significant differences in errors only between specific pairs of racial and ethnic groups. Table 3 shows the post hoc Tukey HSD test results for each COVID-19 case predictive model: the baseline, the baseline enhanced with 1 of the 3 state-of-the-art debiasing approaches, and the baseline enhanced with our proposed method (DemOpts). Each row represents the output of the post hoc test, that is, the difference between the normalized mean PBL error of Group 1 and Group 2 (NormPBL_Group1_ - NormPBL_Group2_). If the difference is positive, it means that the normalized mean predictive error is higher for Group 1; if the difference is negative, the normalized PBL error is higher for Group 2 (superscript b indicates statistically significant differences).

The first relevant observation when examining the table is that the baseline model, focused on predicting COVID-19 county cases with no debiasing approach is highly biased, with statistically significant differences between the mean normalized errors across all pairs of races, except for the comparison between Asian and Black counties as well as Hispanic and White counties, for which there is no statistically significant difference. These results reveal that there is no racial or ethnic group that achieves hard error parity and motivate our exploration of whether state-of-the-art debiasing methods or our proposed DemOpts can improve the hard error parity results of the baseline model.

**Table 2.** ANOVA *F* test statistics comparing mean prediction errors.

Fairness method	F statistic (*df*)
Baseline	1195.398^a^ (3080)
Group	1455.528^a^ (3080)
Individual	1469.698^a^ (3080)
Sufficiency	1195.651^a^ (3080)
DemOpts^b^	668.769^a^ (3080)

a *P*<.001.

b DemOpts: Demographic Optimization.

**Table 3.** Hard error parity analysis. Each value represents the difference between the mean normalized pinball loss for each pair of racial and ethnic groups and indicates whether the difference is statistically significant.

Groups 1 and 2	Baseline	Group	Individual	Sufficiency	DemOpts^a^
Asian					
Black	−0.11	−0.20	−0.12	−0.11	1.32
Hispanic	−2.30^b^	−2.65^b^	−2.50^b^	−2.29^b^	−0.77**^c^**
White	−2.06^b^	−2.51^b^	−2.51^b^	−2.06^b^	−0.96**^c^**
Black					
Hispanic	−2.18^b^	−2.45^b^	−2.38^b^	−2.17^b^	−2.09^b^
White	−1.94^b^	−2.31^b^	−2.39^b^	−1.94^b^	−2.29^b^
Hispanic					
White	0.23	0.14	−0.01	0.23	−0.19

a DemOpts: Demographic Optimization.

b *P*<.001.

c These values denote no significant difference between the prediction errors of Asian and White counties and of Asian and Hispanic counties.

When examining Table 3, we can observe that predictive models enhanced with the individual, group, or sufficiency debiasing methods do not improve the hard error parity over the baseline. On the one hand, similarly to the baseline model, the state-of-the-art debiasing methods (TFT_Individual_, TFT_Group_, and TFT_Sufficiency_) achieve hard error parity between Asian and Black counties and between Hispanic and White counties, that is, the mean error difference between these counties is not significant, pointing to a fair distribution of errors. On the other hand, for each pair of racial and ethnic groups whose prediction error distributions are significantly different for the baseline (rows with asterisks in the Baseline column), they remain significantly different for the individual, group, and sufficiency debiasing methods (rows with superscript b in the individual, group, and sufficiency columns).

When examining the significant mean PBL differences between racial and ethnic groups for the baseline and the state of the art debiasing models, we observe that all coefficients have similar values, signaling similar significant mean PBL differences between racial and ethnic groups (with values between 1.942 and 2.659 error cases per 1000 population). The sign of the coefficients reveals higher mean PBL errors for Hispanic and White counties when compared to Asian or Black counties, and higher mean PBL errors for White counties when compared to Hispanic counties across all models. For example, Hispanic and White counties have mean prediction errors 2.302 and 2.064 cases higher, respectively, when compared to Asian counties and while using the baseline model; and Hispanic and White counties have errors 2.457 and 2.313 cases higher, respectively, when compared to Black counties using the baseline model enhanced with the Group debiasing approach.

Moving on to DemOpts, the table shows that our proposed approach is the only debiasing method that achieves hard error parity in more cases than the baseline, effectively removing some of the associations between race and ethnicity and the normalized mean error distribution (PBL). Specifically, DemOpts removes the significant difference between the prediction errors of Asian and White counties and of Asian and Hispanic counties (refer to values with supercript c in Table 3), effectively achieving hard error parity for Asian counties, that is, the mean PBL in Asian counties is always similar to the mean error in counties of all the other racial and ethnic groups. These improvements occur additionally to hard error parity already seen in TFT_Baseline_ (hard error parity between Asian and Black counties and between Hispanic and White counties), which are also present in the other 3 debiasing methods. In other words, DemOpts improves the hard error parity of case predictions for 2 additional racial and ethnic pairs compared with any of the other debiasing methods.

Finally, when looking specifically at the hard error parity between protected (Asian, Black, and Hispanic) and unprotected groups (White), DemOpts achieves hard error parity for Asian and Hispanic groups; that is, their mean prediction errors are not significantly different from those of White counties, while the baseline and the other 3 debiasing methods only achieve hard error parity for the Hispanic group when compared to White counties. These findings with respect to White counties motivate the evaluation of the soft error parity of the different models to determine, for example, whether DemOpts achieves the best soft error parity for the Black group (since hard error parity was not achieved), or to see if DemOpts has better soft error parity than other debiasing methods for Asian or Hispanic groups. Next, we explore the soft error parity metric for the TFT baseline and for all TFT models enhanced with debiasing approaches.

### Soft Error Parity Results

Table 4 shows the distance to the perfect soft error parity for each of the debiasing approaches across all protected racial and ethnic groups. As we can observe, DemOpts has the smallest values—closest distances to perfect soft error parity—for Asian and Black counties, while the individual debiasing method almost achieves perfect soft error parity for the Hispanic counties. In other words, DemOpts is the debiasing approach that produces the most similar errors between Asian and White counties and between Black and White counties, thereby achieving the largest reduction in predictive bias. On the other hand, the Individual debiasing method achieves errors for Hispanic counties that are closest to the White group. In addition, it is important to highlight that the Group and Sufficiency debiasing methods achieve soft error parities that are close to the TFT_Baseline_, which is not enhanced with any debiasing method.

**Table 4.** Soft error parity analysis. Each value represents the distance (|1-AER_race_|) for each protected group and debiasing method. TFT_DemOpts_ achieves the highest soft error parity for 2 of the 3 protected races under study.

Group	Baseline	Group	Individual	Sufficiency	DemOpts^a^
Asian	0.811	0.842	0.850	0.811	0.454^b^
Black	0.764	0.774	0.807	0.764	0.681^b^
Hispanic	0.093	0.048	0.003^b^	0.093	0.12

a DemOpts: Demographic Optimization.

b Smallest error parity for the particular group

Overall, these results reveal that DemOpts is the debiasing approach that improves the soft error parity of case prediction models, with errors for Asian and Black counties being the closest to errors in White counties. When accounting for additional factors, DemOpts outperforms the other methods by reducing the racial associations of model error.

In Table S1 in Multimedia Appendix 1, we provide and discuss the results for the quantile regression analysis in detail. Overall, the results confirm our findings with majority race labels, with DemOpts consistently outperforming other methods, showing the smallest coefficient magnitude for associations between the percentage of Asian, Black, and Hispanic populations and model error.

## Discussion

### Principal Findings

Through our comparison of model performance for COVID-19 case prediction across counties of differing racial demographics, we showed that DemOpts outperforms other baselines for debiasing predictions. In our analysis of hard error parity, we found that DemOpts was the only debiasing method to eliminate statistically significant relationships between prediction error and racial demographics when compared with the baseline. While some significant associations remained, DemOpts achieved hard error parity for Asian vs White counties and Asian vs Hispanic counties. In the soft error parity analysis, DemOps substantially outperformed the baselines for Asian and Black counties, with a 69.4% reduction and 23% reduction, respectively, compared with the next closest method.

### Why is DemOpts Better?

The results showed that DemOpts is the only debiasing approach to achieve both hard and soft error parity for all 3 racial minority groups when compared with White counties.

In an attempt to understand why DemOpts succeeds in increasing both hard and soft error parity in the context of COVID-19 county case predictions, and compared with other debiasing methods, we computed the average PBL for each racial and ethnic group and for each predictive model enhanced, or not, with a debiasing method (refer to Table 5). We observed that DemOpts achieves better hard and soft error parity metrics because it considerably increases the errors for Asian and Black counties with respect to the baseline, until the differences with Hispanic and White are made not statistically significant (hard error parity) or closer to the White mean errors (soft error parity). Comparing Tables 4 and 5, we observed that DemOpts achieves considerably higher fairness for the Hispanic group (when compared to White) than for the Asian and Black groups (0.12 vs 0.454 and 0.681 in Table 4). As a result, the average PBL error for the Hispanic group (3.59 in Table 5) is considerably higher than the Asian and Black racial groups (1.7 and 1, respectively). We hypothesize that the differences in average errors and performance across racial and ethnic groups could be due to differences in the bias present in the training data, that is, mobility data or COVID-19 case counts could be more biased for Asian or Black groups, thus making it harder to achieve fair predictions when compared to White, and, in turn, due to the fairness-accuracy trade-off, making them more accurate (lower errors).

**Table 5.** Group-wise pinball loss for each model. Demographic Optimization (DemOpts) has higher average pinball loss compared to the other models. The fairness-accuracy tradeoff leads to slightly larger pinball loss values for DemOpts compared to other methods.

Group	Baseline	Group	Individual	Sufficiency	DemOpts^a^
Asian	0.482	0.472	0.444	0.479	1.741
Black	0.600	0.674	0.570	0.598	1.015
Hispanic	2.784	3.131	2.951	2.776	3.597
White	2.546	2.987	2.961	2.540	3.192

a DemOpts: Demographic Optimization.

**Figure 6.**
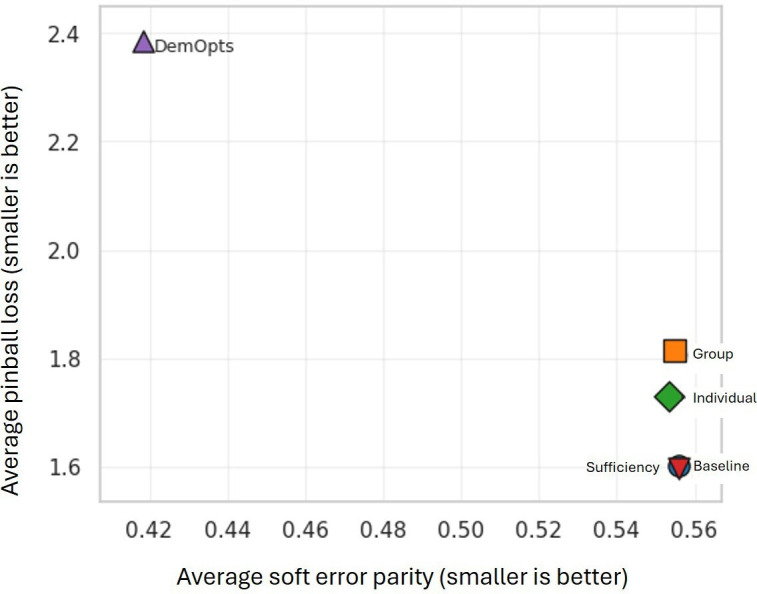
Fairness-accuracy tradeoff. Model error (average pinball loss) vs average soft error parity (|1-AER|) for each model.

These results show that DemOpts’ optimization could not decrease prediction errors while trying to improve fairness, showing a fairness-accuracy trade-off that has been reported previously in the literature [84]. To further clarify this finding, Figure 6 shows both the average PBL and soft parity across all the models considered in this paper. As shown, DemOpts has the lowest soft error parity, but the highest PBL (top-left corner in the plot), while the other models decrease the PBL by sacrificing fairness (higher error parity in the bottom-right corner).

### Limitations

While DemOpts outperforms other state-of-the-art approaches in debiasing COVID-19 predictions, there are some limitations to DemOpts and our evaluation. First, DemOpts is unable to remove all statistical associations for the hard parity analysis, potentially because doing so would impose further reductions in model performance. For the soft parity analysis, the individual fairness approach is best for Hispanic counties, but the difference in parity levels is small. Regarding evaluation, our focus is exclusively on COVID-19 county case prediction—while evaluation on other datasets and prediction tasks would be helpful for future work, our current evaluation provides sufficient evidence to show its applicability to other contexts. In addition, we compare DemOpts to baselines only on error parity metrics. Other fairness metrics may apply to the COVID-19 context and should be evaluated in future work, but we focus on error parity because DemOpts is specifically designed to mitigate it. Finally, we only compare DemOpts and baseline debiasing approaches within TFT models—future work should compare with other commonly used models for COVID-19 case prediction.

Regardless, our novel debiasing approach shows that hard and soft error parity across protected and unprotected racial and ethnic groups can improve relative to other state-of-the-art approaches.

Finally, it is important to clarify that although in this paper, DemOpts focuses on bias mitigation in COVID-19 forecasting, it could also be applied to other health forecasting tasks where sampling bias in data collection can lead to bias in downstream tasks, for example, forecasting flu cases. These forecasts, when done at the county level and when using mobility data to model human spread, could benefit from the DemOpts method by reducing the effect of mobility bias or case count bias on other infectious diseases.

### Conclusion

Researchers have worked tirelessly on the creation of accurate COVID-19 case prediction models to support resource allocation and decision-making. However, sampling and underreporting biases in the data used to train these models have resulted in worse prediction performance for certain protected attributes, pointing to a lack of COVID-19 predictive fairness that could affect decision-making. In this paper, we show that state-of-the-art architectures in COVID-19 case predictions (TFT models) incur unfair prediction error distributions, and we design a novel debiasing approach and evaluation method to increase the fairness of predictions in the context of COVID-19 county case forecasts. DemOpts modifies the loss function in deep learning models to reduce the dependencies between error distributions and racial and ethnic labels. Our results show that DemOpts improves both the hard and soft error parity of COVID-19 county case predictions when compared with state-of-the-art debiasing methods.

## Supplementary material

10.2196/78235 Multimedia Appendix 1 Details on the differentiability and regression analysis of fairness method errors.

## Abbreviations

AER: Accuracy Equity Ratio
CDC: Centers for Disease Control and Prevention
HSD: honestly significant difference
NYT: New York Times
OD: origin-destination
PBL: pinball loss
TFT: temporal fusion transformer

## References

[1] (2023). COVID-19 forecasting and mathematical modeling. Centers for Disease Control and Prevention (CDC).

[2] (2020). COVID-19 Forecast Hub.

[3] Zou D, Wang L, Xu P, Chen J, Zhang W, Gu Q (2020). Epidemic model guided machine learning for COVID-19 forecasts in the United States. medRxiv.

[4] Pei S, Shaman J (2020). Initial simulation of SARS-CoV2 spread and intervention effects in the continental US. medRxiv.

[5] Chiang WH, Liu X, Mohler G (2020). Hawkes process modeling of COVID-19 with mobility leading indicators and spatial covariates. medRxiv.

[6] Galasso J, Cao DM, Hochberg R (2022). A random forest model for forecasting regional COVID-19 cases utilizing reproduction number estimates and demographic data. Chaos Solitons Fractals.

[7] Arik S, Li CL, Yoon J, Sinha R, Epshteyn A, Le L (2020). Interpretable sequence learning for COVID-19 forecasting. arXiv.

[8] Zhang-James Y, Hess J, Salkin A (2021). A seq2seq model to forecast the COVID-19 cases, deaths and reproductive R numbers in US counties. Res Sq.

[9] Le M, Ibrahim M, Sagun L, Lacroix T, Nickel M (2021). Neural relational autoregression for high-resolution COVID-19 forecasting. https://epidamik.github.io/2021/papers/epiDAMIK_40_paper_12.pdf.

[10] Lucas B, Vahedi B, Karimzadeh M (2023). A spatiotemporal machine learning approach to forecasting COVID-19 incidence at the county level in the USA. Int J Data Sci Anal.

[11] Angulo FJ, Finelli L, Swerdlow DL (2021). Estimation of US SARS-CoV-2 infections, symptomatic infections, hospitalizations, and deaths using seroprevalence surveys. JAMA Netw Open.

[12] Times TNY (2020). Coronavirus (covid-19) data in the United States. GitHub.

[13] Dong E, Du H, Gardner L (2020). An interactive web-based dashboard to track COVID-19 in real time. Lancet Infect Dis.

[14] Smittenaar P, Stewart N, Sutermaster S (2021). A COVID-19 community vulnerability index to drive precision policy in the US. medRxiv.

[15] (2022). COVID-19 community mobility reports. https://www.google.com/covid19/mobility/.

[16] (2022). Mobility trends reports. https://covid19.apple.com/mobility.

[17] Gursoy F, Kakadiaris IA (2022). Error parity fairness: testing for group fairness in regression tasks. arXiv.

[18] Erfani A, Frias-Martinez V (2023). A fairness assessment of mobility-based COVID-19 case prediction models. PLoS ONE.

[19] Del Rios M, Puente S, Vergara-Rodriguez P, Sugrue N (2022). Latino invisibility in the pandemic. AMA J Ethics.

[20] Coston A, Guha N, Ouyang D, Lu L, Chouldechova A, Ho DE Leveraging administrative data for bias audits: assessing disparate coverage with mobility data for COVID-19 policy.

[21] Meraihi Y, Gabis AB, Mirjalili S, Ramdane-Cherif A, Alsaadi FE (2022). Machine learning–based research for COVID-19 detection, diagnosis, and prediction: a survey. SN Comput Sci.

[22] Lim B, Arık SÖ, Loeff N, Pfister T (2021). Temporal fusion transformers for interpretable multi-horizon time series forecasting. Int J Forecast.

[23] Hochreiter S, Schmidhuber J (1997). Long short-term memory. Neural Comput.

[24] Velickovic P, Cucurull G, Casanova A, Romero A, Lio P, Bengio Y (2017). Graph attention networks stat. Stat (Int Stat Inst).

[25] Yu B, Yin H, Zhu Z Spatio-temporal graph convolutional networks: a deep learning framework for traffic forecasting.

[26] Duan L, Hu T, Cheng E, Zhu J, Gao C Deep convolutional neural networks for spatiotemporal crime prediction. https://www.semanticscholar.org/paper/Deep-Convolutional-Neural-Networks-for-Crime-Duan-Hu/407b84eb7ecfea0557b1a3e38ff0767b9bf25622.

[27] Li S, Jin X, Xuan Y, Zhou X, Chen W, Wang YX, Wallach H, Larochelle H, Beygelzimer A, d’ ABF, Fox E, Garnett R Enhancing the locality and breaking the memory bottleneck of transformer on time series forecasting. https://proceedings.neurips.cc/paper_files/paper/2019/file/6775a0635c302542da2c32aa19d86be0-Paper.pdf.

[28] Zhou H, Zhang S, Peng J (2020). Informer: beyond efficient transformer for long sequence time-series forecasting. arXiv.

[29] Wu H, Xu J, Wang J, Long M (2021). Autoformer: decomposition transformers with auto-correlation for long-term series forecasting. arXiv.

[30] Zhou T, Ma Z, Wen Q, Wang X, Sun L, Jin R (2022). FEDformer: frequency enhanced decomposed transformer for long-term series forecasting. arXiv.

[31] Liu S, Yu H, Liao C, Li J, Lin W, Liu AX Pyraformer: low-complexity pyramidal attention for long-range time series modeling and forecasting. https://openreview.net/forum?id=0EXmFzUn5I.

[32] Nie YH, Sinthong P, Kalagnanam J, Nguyen N (2023). A time series is worth 64 words: long-term forecasting with transformers. arXiv.

[33] Vieira MR, Frías-Martínez E, Bakalov P, Frías-Martínez V, Tsotras VJ Querying spatio-temporal patterns in mobile phone-call databases.

[34] Hernandez M, Hong L, Frias-Martinez V, Whitby A, Frias-Martinez E (2017). Estimating poverty using cell phone data: evidence from guatemala. https://documents.worldbank.org/en/publication/documents-reports/documentdetail/122541487082260120/estimating-poverty-using-cell-phone-data-evidence-from-guatemala.

[35] Frias-Martinez V, Virseda J (2013). Cell phone analytics: Scaling human behavior studies into the millions. Information Technologies & International Development.

[36] Rubio A, Frias-Martinez V, Frias-Martinez E, Oliver N Human mobility in advanced and developing economies: a comparative analysis. http://www.aaai.org/ocs/index.php/SSS/SSS10/paper/view/1095.

[37] Wu J, Frias-Martinez E, Frias-Martinez V (2021). Spatial sensitivity analysis for urban hotspots using cell phone traces. Environ Plan B: Urban Anal City Sci.

[38] Wu J, Abrar SM, Awasthi N, Frias-Martinez E, Frias-Martinez V (2022). Enhancing short-term crime prediction with human mobility flows and deep learning architectures. EPJ Data Sci.

[39] Wu J, Abrar SM, Awasthi N, Frías-Martínez V (2023). Auditing the fairness of place-based crime prediction models implemented with deep learning approaches. Comput Environ Urban Syst.

[40] Wesolowski A, Eagle N, Tatem AJ (2012). Quantifying the impact of human mobility on malaria. Science.

[41] Hong L, Fu C, Torrens P, Frias-Martinez V (2017). Understanding citizens’ and local governments’ digital communications during natural disasters. WebSci ’17: Proceedings of the 2017 ACM on Web Science Conference.

[42] Isaacman S, Frias-Martinez V, Frias-Martinez E (2018). Modeling human migration patterns during drought conditions in La Guajira, Colombia. COMPASS ’18: Proceedings of the 1st ACM SIGCAS Conference on Computing and Sustainable Societies.

[43] Bengtsson L, Gaudart J, Lu X (2015). Using mobile phone data to predict the spatial spread of cholera. Sci Rep.

[44] Ghurye J, Krings G, Frias-Martinez V A framework to model human behavior at large scale during natural disasters.

[45] Hong L, Frias-Martinez V (2020). Modeling and predicting evacuation flows during hurricane Irma. EPJ Data Sci.

[46] Frias-Martinez V, Virseda J, Frias-Martinez E (2010). Socio-economic levels and human mobility. Qual Meets Quant Workshop-QMQ.

[47] Fu C, McKenzie G, Frias-Martinez V, Stewart K (2018). Identifying spatiotemporal urban activities through linguistic signatures. Comput Environ Urban Syst.

[48] Frias-Martinez V, Virseda J, Gomero A (2012). Mobilizing education: evaluation of a mobile learning tool in a low-income school. MobileHCI ’12: 14th International Conference on Human Computer Interaction with Mobile Devices and Services.

[49] Hong L, Frias-Martinez E, Frias-Martinez V (2016). Topic Models to Infer Socio-Economic Maps. AAAI.

[50] Frias-Martinez V, Soto V, Virseda J, Frias-Martinez E (2012). Computing cost-effective census maps from cell phone traces. Department of Computer Science, Colombia University.

[51] Kang W, Oshan T, Wolf LJ (2019). A roundtable discussion: defining urban data science. Environ Plan B Urban Anal City Sci.

[52] Hong L, Wu J, Frias-Martinez E, Villarreal A, Frias-Martinez V Characterization of internal migrant behavior in the immediate post-migration period using cell phone traces.

[53] Frias-Martinez V, Sloate E, Manglunia H, Wu J (2021). Causal effect of low-income areas on shared dockless e-scooter use. Transp Res D Trans Environ.

[54] Badr HS, Gardner LM (2021). Limitations of using mobile phone data to model COVID-19 transmission in the USA. Lancet Infect Dis.

[55] Tsai TC, Arik S, Jacobson BH (2022). Algorithmic fairness in pandemic forecasting: lessons from COVID-19. NPJ Digit Med.

[56] Abrar SM, Awasthi N, Smolyak D, Frias-Martinez V (2023). Analysis of performance improvements and bias associated with the use of human mobility data in COVID-19 case prediction models. ACM J Comput Sustain Soc.

[57] Douglas MD, Respress E, Gaglioti AH (2021). Variation in reporting of the race and ethnicity of COVID-19 cases and deaths across US states: April 12, 2020, and November 9, 2020. Am J Public Health.

[58] Estiri H, Strasser ZH, Rashidian S (2022). An objective framework for evaluating unrecognized bias in medical AI models predicting COVID-19 outcomes. J Am Med Inform Assoc.

[59] Gross CP, Essien UR, Pasha S, Gross JR, Wang SY, Nunez-Smith M (2020). Racial and ethnic disparities in population-level Covid-19 mortality. J Gen Intern Med.

[60] Agarwal A, Dudík M, Wu ZS (2019). Fair regression: quantitative definitions and reduction-based algorithms. arXiv.

[61] Fitzsimons J, Al Ali A, Osborne M, Roberts S (2019). A general framework for fair regression. Entropy (Basel).

[62] Brunet ME, Alkalay-Houlihan C, Anderson A, Zemel R (2018). Understanding the origins of bias in word embeddings. arXiv.

[63] Calmon FP, Wei D, Ramamurthy KN, Varshney KR (2017). Optimized data pre-processing for discrimination prevention. arXiv.

[64] Yan B, Seto S, Apostoloff N (2022). FORML: learning to reweight data for fairness. arXiv.

[65] Wang Y, Singh L (2023). Mitigating demographic bias of machine learning models on social media.

[66] Yang J, Soltan AAS, Clifton DA (2022). Algorithmic fairness and bias mitigation for clinical machine learning: a new utility for deep reinforcement learning. medRxiv.

[67] Das R, Dooley S (2023). Fairer and more accurate tabular models through NAS. arXiv.

[68] Jagodnik KM, Ray F, Giorgi FM, Lachmann A (2020). Correcting under-reported COVID-19 case numbers: estimating the true scale of the pandemic. medRxiv.

[69] Albani V, Loria J, Massad E, Zubelli J (2021). COVID-19 underreporting and its impact on vaccination strategies. BMC Infect Dis.

[70] Berk R, Heidari H, Jabbari S, Joseph M, Kearns M, Morgenstern J (2017). A convex framework for fair regression. arXiv.

[71] Shah A, Bu Y, Lee JK, Das S, Panda R, Sattigeri P, Chaudhuri K, Jegelka S, Song L, Szepesvari C, Niu G, Sabato S (2022). Selective regression under fairness criteria. https://proceedings.mlr.press/v162/shah22a.html.

[72] Cramer EY, Huang Y, Wang Y (2022). The United States COVID-19 Forecast Hub dataset. Sci Data.

[73] Kader F, Smith CL (2021). Participatory approaches to addressing missing COVID-19 race and ethnicity data. Int J Environ Res Public Health.

[74] Blanca MJ, Alarcón R, Arnau J, Bono R, Bendayan R (2017). Non-normal data: is ANOVA still a valid option?. Psicothema.

[75] Zimmerman DW (1987). Comparative power of student t test and Mann-Whitney U test for unequal sample sizes and variances. The Journal of Experimental Education.

[76] Castelnovo A, Crupi R, Greco G, Regoli D, Penco IG, Cosentini AC (2022). A clarification of the nuances in the fairness metrics landscape. Sci Rep.

[77] Kang Y, Gao S, Liang Y, Li M, Rao J, Kruse J (2020). Multiscale dynamic human mobility flow dataset in the U.S. during the COVID-19 epidemic. Sci Data.

[78] Schlosser F, Sekara V, Brockmann D, Garcia-Herranz M (2021). Biases in human mobility data impact epidemic modeling. arXiv.

[79] Mapbox. https://www.mapbox.com/about/maps.

[80] OpenStreetMap. https://www.openstreetmap.org/about/.

[81] Improve this map. https://apps.mapbox.com/feedback/.

[82] (2020). PyTorch-based forecasting with sktime integration sktime.

[83] Gatalo O, Tseng K, Hamilton A, Lin G, Klein E, CDC MInD-Healthcare Program (2021). Associations between phone mobility data and COVID-19 cases. Lancet Infect Dis.

[84] Kim JS, Chen J, Talwalkar A (2020). FACT: a diagnostic for group fairness trade-offs. ICML’20: Proceedings of the 37th International Conference on Machine Learning.

