# Supplementary material for: Fairness Correction in COVID-19 Predictive Models Using Demographic Optimization: Algorithm Development and Validation Study
Source: Online J Public Health Inform. 2026 Feb 3;18:e78235. doi: 10.2196/78235 (PMC12866456; doi:10.2196/78235)
Supplement: Multimedia Appendix 1 [file ojphi-v18-e78235-s001.pdf]

Fairness Correction in COVID-19 Predictive Models Using DemOpts: Algorithm Development and Validation

Appendix

S.1 DemOpts Method

Description of the Regression Task

Let $\{CC_{c,t}\}_{c,t=0}^{C,T}$ be the COVID-19 case count time series, where $CC_{c,t}$ represents the COVID-19 case count for county c on day t. Similarly, we define $\{Mob_{c,t}\}_{c,t=0}^{C,T}$ for mobility data. We convert static ethnicity data into time series by repeating the same values $\{Eth_{c,t}\}_{c,t=0}^{C,T}$. The forecasting task can be defined as:

$$
\begin{aligned}
CC_{c,T+1:T+h} \; = \; f(&[CC_{c,i}, Mob_{c,i}, Eth_{c,i}],\\
&[CC_{c,i+1}, Mob_{c,i+1}, Eth_{c,i+1}],\\
&[CC_{c,i+2}, Mob_{c,i+2}, Eth_{c,i+2}],\\
&\ldots\ldots\\
&[CC_{c,T}, Mob_{c,T}, Eth_{c,T}],)
\end{aligned}
$$

where:
- $CC_{c,T+1:T+h}$ is the forecast for 1 to h time steps ahead. In our experiments h = 45.
- $f(\cdot)$ is the TFT forecasting network.

Loss function

We define adjusted loss as a re-scaled version of the original loss. We consider the association (regression coefficients and *p*-value) between race percentage of a county, the percentage of population for each race in the county and the PBL of the current model on that county.

$$L_{adj} = PBL(y_{ip}, y_i) + \sum_j H(pval_j)(|\beta_j| * D_j * L) \quad where$$

$$H(x) = \begin{cases} 1, & if\ x < 0.005 \\ 0, & if\ x \geq 0.005 \end{cases}$$

Thus, loss adjustment factor K will be

$$K \; = \sum_j H(pval_j)(|\beta_j| * D_j * L)$$

In the ideal fair scenario, $H(pval_j) = 0$ or $\beta_j = 0$ i.e. the coefficient (β) would be 0 or, *p*-value (pval) of the coefficient would be a larger number (Thus $H(pval) = 0$). In all other cases, the findings show an association between our forecasting model (TFT) and the race/ethnic population of the US counties.

Differentiability of Loss function

We compute the adjusted loss by utilizing the coefficients and p-values derived from regression models. The regression coefficients are computed separately, and the process is detached from the autograd (PyTorch's automatic differentiation engine). Here, we show how the re-weighting approach changes the backpropagation in the training.

Let's assume an input X to the model. The output Y and $\mathrm{PBL}(y_j, y_{truth})$ represents the PBL for the output. Computing the gradient with respect to the last layer (w_n), we get:

$$\nabla_{w_n} L_{adj} = \nabla_{w_n} PBL(y_j, y_{truth}) + \nabla_{w_n} K$$

$$\nabla_{w_n} K = \sum_{race=j} H\left(pval_j\right) \cdot |\beta_j| \cdot D_j \cdot \nabla_{w_n} PBL(y_{ip}, y_i)$$

where all the adjustment factors are constants multiplied by the PBL loss function (which is a function of prediction and ground truth). Thus, the gradient is scaled based on the regression coefficients of the n-th layer, in this example. Similarly, one can see that for any previous (n-1 layer), the gradient would be multiplied by the same factor. Eg. for $n - 2^{th}$ layer the adjusted factor would be:

$$\nabla_{w_{n-2}} L_{adj} = \nabla_{w_{n-2}} PBL(y_j, y_{truth}) + \nabla_{n-2} K$$

Thus, the new proposed DemOpts method is differentiable and applicable for gradient based optimization approaches like deep neural networks.

S.2 Fairness Regression analysis

Regression Analysis of model errors

While majority-based race labels for counties are standard in literature[1,2], in our analyses there are very few minority labelled counties which could lead to questions on the statistical significance of the results. To assess that, we run a quantile regression analysis over each of the model errors for each county and their demographic composition. The formula in R is as follows:

$$normed\_abs\_error \sim pct\_asian + pct\_hispanic + pct\_black + urban\_rural + forecast\_period$$

where normed_abs_error is the normalized error for the county forecast, $pct\_\{Asian, black, hispanic\}$ are the demographic features for the corresponding county, and urban/rural are county classification according to National Center for Health Statistics[1] . Forecast period is the day number for which the forecast was done

[1] https://www.cdc.gov/nchs/data-analysis-tools/urban-rural.html

for the model (each county has 52-day forecast window). Quantile regression is robust to outliers and COVID-19 forecasting errors are not normally distributed in the data. Thus, median is a better representation of the model errors, and we use quantile regression for this regression analysis.

Table S.1 shows the magnitude of the regression coefficients highlighting the association between model error and racial/ethnic composition of the county. For DemOpts we show multiple rows. Each row represents the coefficients for different internal p-value hyperparameter cutoff points. The details of this ablation study are presented in the next section. The important analysis here is that DemOpts leads to the smallest error associations with demographic composition of a county throughout all minority groups, confirming prior analyses presented in this paper. This demonstrates that our proposed DemOpts methodology outperforms other existing methods in terms of fairness.

Ablation study: Hyperparameter p-value insde DemOpts

We also perform an ablation study on the hyperparameter "pval" inside DemOpts' optimization. We run DemOpts on several different values (0.5, 0.1, 0.05, 0.01, 0.005, 0.001) and compare the AER of the models (Table S.1) under DemOpts (last six rows in Table S.1). The coefficients indicate the association between model error and demographic composition. We do not aim for the lowest coefficient (i.e. -∞), rather we seek a model with the smallest absolute relation between demographic composition and model error. This would help us achieve the model which is fair for all races/ethnicity. As Table S.1 shows, DemOpts achieves the smallest associations with the error compared to all other models, with pval thresholds between 0.1 and 0.005. Smaller pval thresholds generally lead to smaller associations, although the distribution changes across racial and ethnic groups, pointing to distinct optimal p-values. This could indicate that using stricter pval requirements (i.e. loss adjustment when there are stronger significant associations between model predictions and county demographics) to adjust the loss in DemOpts leads to fairer deep learning models.

Table S1: Magnitude of regression coefficients for association between race percentage and normalized model error across counties (closer to 0 is better i.e. no association between model error and demographic characteristics). All regression coefficients reported with coefficient significance (P<.001). ***Bold*** *highlights the best value in each column.*

	abs(Demographic attribute coefficient)		
Model	% Asian	% Black	% Hispanic
Baseline (mob, demo, no fair)	0.001859	0.000919	0.000233
Individual	0.001872	0.001152	0.000302
Group	0.00193	0.001045	0.000238
Sufficiency	0.001864	0.000928	0.000244
**DemOpts**			
Pval Threshold:0.5	0.002204	0.001349	0.000368
Pval Threshold :0.1	0.001633	0.000741	**1.8e-05**

Pval Threshold :0.05	0.001834	**8.7e-05**	0.000168
Pval Threshold :0.01	0.002034	0.00148	0.000445
Pval Threshold :0.005	**0.001485**	0.001352	0.000373
Pval Threshold :0.001	0.001619	0.000774	0.000138

References

1. Coston A, Guha N, Ouyang D, Lu L, Chouldechova A, Ho DE. Leveraging Administrative Data for Bias Audits: Assessing Disparate Coverage with Mobility Data for COVID-19 Policy. In: Proceedings of the 2021 ACM Conference on Fairness, Accountability, and Transparency [Internet]. New York, NY, USA: Association for Computing Machinery; 2021. p. 173–84. (FAccT '21). Available from: https://doi.org/10.1145/3442188.3445881

2. Abrar SM, Awasthi N, Smolyak D, Frias-Martinez V. Analysis of performance improvements and bias associated with the use of human mobility data in COVID-19 case prediction models. ACM Journal on Computing and Sustainable Societies. 2023. Available from: https://doi.org/10.48550/arXiv.2407.10304
